# Trust your guts? The effect of gut section on diet composition and impact of *Mus musculus* on islands using metabarcoding

**DOI:** 10.1002/ece3.8638

**Published:** 2022-03-07

**Authors:** Catarina J. Pinho, Evandro P. Lopes, Joana Paupério, Isildo Gomes, Maria M. Romeiras, Raquel Vasconcelos

**Affiliations:** ^1^ CIBIO Centro de Investigação em Biodiversidade e Recursos Genéticos, InBIO Laboratório Associado, Campus de Vairão, Universidade do Porto Vairão Portugal; ^2^ Departamento de Biologia Faculdade de Ciências da Universidade do Porto Porto Portugal; ^3^ BIOPOLIS Program in Genomics, Biodiversity and Land Planning CIBIO, Campus de Vairão Vairão Portugal; ^4^ ISECMAR‐UTA Instituto Engenharia e Ciências do Mar da Universidade Técnica do Atlântico Mindelo Cabo Verde; ^5^ INIDA Instituto Nacional de Investigação e Desenvolvimento Agrário Santiago Cabo Verde; ^6^ LEAF‐ISA Linking Landscape, Environment, Agriculture and Food, Instituto Superior de Agronomia Universidade de Lisboa Lisboa Portugal

**Keywords:** Cabo Verde Islands, diet, gastrointestinal tract, house mouse, invasive species, invertebrates, next‐generation sequencing, plants

## Abstract

1. DNA metabarcoding is widely used to characterize the diet of species, and it becomes very relevant for biodiversity conservation, allowing the understanding of trophic chains and the impact of invasive species. The need for cost‐effective biodiversity monitoring methods fostered advances in this technique. One question that arises is which sample type provides a better diet representation.
2. Therefore, with this study, we intended to evaluate if there were differences in diet estimates according to the section of the gastrointestinal tract analysed and which section(s) provided the best diet representation. Additionally, we intended to infer the ecological/economic impacts of an invader as a model of the potential effects in an originally mammal‐free ecosystem.
3. We examined the gut contents of the house mouse *Mus musculus* introduced to Cabo Verde, considering three sections: stomach, small intestine, and large intestine. We applied a DNA‐metabarcoding approach using two genetic markers, one specific for plants and another for invertebrates.
4. We showed that this invader consumed 131 taxa (73 plants and 58 invertebrates). We obtained significant differences in the composition of two of the three sections, with a higher incidence of invertebrates in the stomach and plants in the intestines. This may be due to stomach inhibitors acting on plants and/or to faster absorption of soft‐body invertebrates compared to the plant fibers in the intestines. We verified that the impact of this invader in the ecosystem is predominantly negative, as at least 50% of the ingested items were native, endemic, or economically important taxa, and only 19% of the diet items were exotics.
5. Overall, results showed the need to analyse only two gastrointestinal tract sections to obtain robust diet data, increasing the cost‐effectiveness of the method. Furthermore, by uncovering the native taxa most frequently preyed on by mice, this DNA‐metabarcoding approach allowed us to evaluate efficiently which are at the highest risk.

## Graphical abstract

In this article, we assessed the differences in diet estimates and potential ecological and economic impact measurements according to the section of the gastrointestinal tract (stomach, small and large intestine) analysed. We aimed to test which section(s) provided a better diet representation and putative impact of a common inland invader. For that, we examined the gut contents of house mice *Mus musculus* introduced to Cabo Verde Islands, applying a DNA‐metabarcoding approach. Our results demonstrated that with only two or the three gut sections it is possible to obtain robust diet and impact representations, and that there is a higher incidence of invertebrates in the stomach and of plants in the intestines. In addition, we were able to demonstrate efficiently and with much higher taxonomic resolution than using traditional methods that the impact of this invader is predominantly negative.

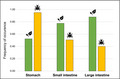

## Abstract in Portuguese

O *metabarcoding* de ADN é amplamente utilizado para a caracterização da dieta de espécies, e tornou‐se bastante relevante para a conservação da biodiversidade, permitindo a compreensão sobre cadeias tróficas e o impacto de espécies invasoras. A necessidade de métodos de monitorização da biodiversidade com uma boa relação custo‐benefício fomentaram avanços nesta técnica. Uma questão que se coloca é qual o tipo de amostra que fornece uma melhor representação da dieta. Deste modo, com este estudo, pretendemos avaliar se existem diferenças nas estimativas da dieta de acordo com a secção do tracto gastrointestinal analisada e qual(is) a(s) secção(ões) que proporciona(m) uma melhor representação da dieta. Adicionalmente, pretendemos inferir os impactos ecológicos/ económicos de um invasor como um modelo dos efeitos potenciais que este pode ter num ecossistema originalmente sem mamíferos. Analisámos os conteúdos gastrointestinais do rato doméstico *Mus musculus* introduzido em Cabo Verde, considerando três secções: estômago, intestino delgado e intestino grosso. Aplicámos uma abordagem de *metabarcoding* de ADN usando dois marcadores genéticos, um específico para plantas e outro para invertebrados. Mostrámos que este invasor consumiu 131 taxa (73 plantas e 58 invertebrados). Obtivemos diferenças significativas na composição de duas das três secções, com maior incidência de invertebrados no estômago e de plantas nos intestinos. Isto pode dever‐se a inibidores estomacais que agem sobre as plantas e/ ou à absorção mais rápida de invertebrados de corpo mole em comparação com as fibras vegetais nos intestinos. Verificámos que o impacto deste invasor no ecossistema é predominantemente negativo, pois pelo menos 50% dos itens ingeridos eram nativos, endémicos ou economicamente importantes e apenas 19% dos itens da dieta eram exóticos. De modo geral, os resultados mostraram a necessidade de analisar apenas duas secções do tracto gastrointestinal para obter dados robustos da dieta, aumentando a relação custo‐eficácia deste método. Além disso, ao descobrir os taxa nativos mais frequentemente predados por ratos, a abordagem de *metabarcoding* de ADN permitiu‐nos avaliar com eficiência quais estão sob maior risco.

## 1 INTRODUCTION

Dietary studies can reveal valuable information on how species exploit the available food resources and intervene in ecological processes (Siegenthaler et al., 2019). Several methods can be implemented to characterize diet, such as direct observation, morphological identification, and stable isotopes analysis (Margalida et al., 2005; Martín et al., 2017; Symondson, 2002). However, with the recent generalization of high‐throughput sequencing methodologies, the identification of prey items was further improved (Pompanon et al., 2012). DNA‐metabarcoding, combined with the power of next‐generation sequencing (NGS) technologies (Shendure & Ji, 2008), became widely used to characterize species diets (Taberlet et al., 2012). DNA‐based methodologies allow the identification of prey material even when hard parts cannot be recovered after digestion, contrary to other methods. These provide comprehensive taxonomic identification of diet items within highly diverse diets, relying less on taxonomic expertise, and can be applied to noninvasive or degraded samples (Pompanon et al., 2012). The need for cost‐effective biodiversity monitoring methods fostered the advances of this technique, becoming highly relevant for biodiversity conservation, by allowing the understanding of trophic chains, and ultimately the ecological impact of invasive species (Westfall et al., 2020). Assessment of predation by invaders using DNA‐based approaches is rapidly growing, permitting wildlife managers to identify the most threatened taxa (Harms‐Tuohy et al., 2016; Robeson et al., 2018).

Samples used to characterize diets using DNA‐metabarcoding are generally fecal, or gut contents (Jakubavičiūtė et al., 2017; Thuo et al., 2019). Fecal samples are advantageous if applied to threatened species since they can be obtained with minimum or no impact on individual fitness (Ferreira et al., 2018). However, in comparison with faeces, DNA from gut contents is usually of superior quality and thus commonly used in diet assessments of invaders (Robeson et al., 2018; Siegenthaler et al., 2019). Yet, most studies disregard the contents of the intestines. The detectability of prey DNA in mammalian stomachs versus faeces was already explored, indicating that sample type influenced its duration but not its quality (Egeter et al., 2015). However, to our knowledge, no study explored how analyzing different gastrointestinal tract sections (stomach, large and small intestines), through DNA‐metabarcoding, influences the detectability of diet items. This is particularly important to omnivorous species that consume items with different tissue cell density, digestion rate, and DNA quality decay after digestion (Pompanon et al., 2012).

Invasive species are one of the main causes of biodiversity loss (Butchart et al., 2010), particularly on islands. On geologically young oceanic islands, typically no native terrestrial mammals occur and, in most cases, non‐volant land mammals have been anthropogenically introduced (Whittaker et al., 2017). These introductions already led to the loss of several island endemics worldwide (Doherty et al., 2016). The most widely introduced mammal species to islands are cats *Felis catus* L. and commensal rodents (Doherty et al., 2016). Norway rats *Rattus norvegicus* (Berkenhout 1769), black rats *Rattus rattus* L., Pacific rats *Rattus exulans* (Peale 1848), and house mouse *Mus musculus* L. were unintentional transported on boats to most islands (Jones et al., 2013), causing devastating impacts on fauna and flora. These invaders compromise the stability of native populations by competition for resources, predation, and transmission of diseases (Courchamp et al., 2003; Gaiotto et al., 2020). Furthermore, rodents are the ones causing more damage to the natural patrimony of islands (Russell et al., 2018), leading to great economic losses (Doherty et al., 2017). These omnivorous invaders can have broad impacts on health, culture, and agriculture (Russell et al., 2017). For instance, in Asia, the damage rodents cause to rice crops prevented the feeding of 200 million people (Stenseth et al., 2003). In particular, the house mouse is among the 15 species most prevalent on islands worldwide (Russell et al., 2017). In Australia, mice cause losses of up to 4% of the national agricultural production, equivalent to 40 million dollars in the worst seasons (Singleton, 1997). However, the impacts of these rodents on native and domestic species are rarely quantified, due to the lack of taxonomic knowledge of predated items and slowness in obtaining data using traditional methods of morphological identification.

In Cabo Verde, as in several other geologically young oceanic islands, no indigenous terrestrial mammals occur except for bats (Borloti et al., 2020; Hazevoet & Masseti, 2011). Therefore, all non‐volant terrestrial mammals were introduced during the past 550 years, when the first Europeans arrived in the archipelago. These invaders already caused a great impact contributing to the extinction of endemics, such as the Cabo Verde giant skink *Chioninia coctei* (Duméril & Bibron, 1839), and the extirpation of the giant wall gecko *Tarentola gigas* (Bocage, 1875) on Santa Luzia Island (Mateo et al., 2005; Medina et al., 2021). From the rodent invaders, mice are the most widely distributed occurring across all inhabited and even some uninhabited islands of the archipelago (Hazevoet & Masseti, 2011). Similar to other island ecosystems (Bunce et al., 2009), it is likely that mice have highly negative impacts on Cabo Verdean native flora and agricultural crops. This impact is expected to be higher on more vegetated islands, with higher coverage of both endemic plants and agricultural production, which is the case of Santiago and Santo Antão islands (Brilhante et al., 2021; MAA, 2021). Nevertheless, the damage caused by these invaders in Cabo Verde has never been quantified. With this study, we intend to evaluate if there are differences in diet estimates according to the section of the gastrointestinal tract analysed and which section(s) provide a better representation of the diet and the impacts of an invader using a DNA‐metabarcoding approach. For that, we examined the gut contents of the house mouse introduced to Cabo Verde, considering three sections: stomach, small intestine, and large intestine. Additionally, we aimed to provide a framework to infer the ecological/economic impact of invaders on an island ecosystem, especially in agricultural areas.

## 2 MATERIALS AND METHODS

### 2.1 Study area

**FIGURE 1:**
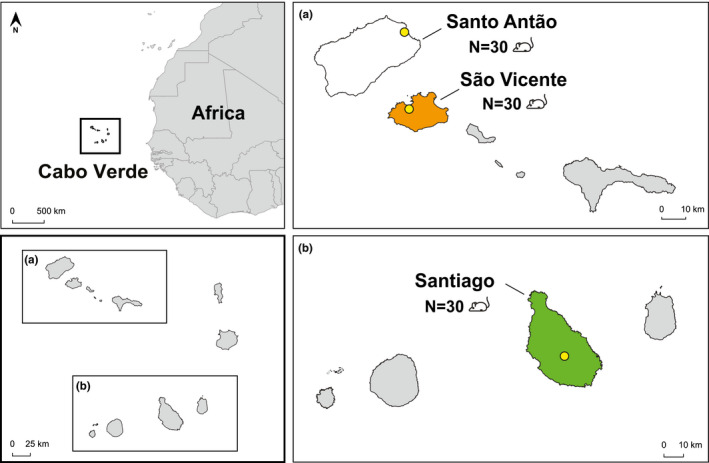
Map of Cabo Verde showing the geographic location of the islands and the sampled sites (yellow circles). (a) Two islands were sampled in the Windward group, Santo Antão (Paúl) and São Vicente (Mindelo). (b) One island was sampled in the Leeward group, Santiago (São Lourenço dos Órgãos). N stands for the number of sequenced samples per island

The Cabo Verde Islands are located in the Atlantic Ocean, approximately 500 km off the African coast. This volcanic archipelago belongs to the biogeographical region of Macaronesia and is composed of ten main islands and several islets (Figure 1). It is politically divided into Windward and Leeward Islands. We sampled two islands of the Windward group (Santo Antão and São Vicente) and one of the Leeward group (Santiago; Figure 1) to have a wider representation of the available diversity of diet items (Arechavaleta et al., 2005).

Santo Antão is the northernmost and second‐largest island of Cabo Verde (Figure 1). With a total area of 779 km², and the second‐largest agricultural area of the archipelago, corresponding to 16% of the national total (Monteiro et al., 2020), mice are expected to be abundant (Hazevoet & Masseti, 2011). The southwestern region of this mountainous island is almost completely arid, while the northeast, where we sampled, receives relatively regular rainfall and thus has more vascular plants and invertebrate endemic richness (Romeiras et al., 2015). São Vicente's total area is 227 km^2^ (Figure 1), and its agricultural areas correspond to only 0.3% of the whole archipelago (Monteiro et al., 2020). Its landscape is composed of stony plains, sandy dunes, and barren hills, as it is very dry with scarce vegetation (Duarte et al., 2008). However, as it harbours one of the largest human settlements of the country (Romeiras et al., 2020), mice are expected to be abundant (Groh, 1982). It is also one of the most important islands considering terrestrial endemic arthropod richness (Lobo & Borges, 2010). Santiago, with 991 km^2^ of total area, is the largest island (Figure 1) and the most important agricultural center of the archipelago (Monteiro et al., 2020). Mice were previously reported on this island in the mountains around São Jorge dos Órgãos and São Domingos (Rabaça & Mendes, 1997). The landscape of Santiago is mostly characterized by steep, tall mountains, although the southeast area is flatter (Neto et al., 2020). Encompassing a wide range of habitats, this island presents the highest number of species and endemism of the archipelago (Duarte et al., 2008; Lobo & Borges, 2010).

### 2.2 Sampling

Sampling took place between the 11th and 27th of November 2019 in the municipalities of Paúl, on Santo Antão, Mindelo, on São Vicente, and São Lourenço dos Órgãos, on Santiago (Figure 1). We placed 40–70 Sherman traps per site in cultivation fields, depending on its size and crop diversity, in groups of 10, distanced by approximately 5 m. We baited each trap with oats, tuna and peanut butter to attract *Mus musculus* individuals. We euthanized the captured mice by cervical dislocation and dissected them for the collection of the gastrointestinal tracts with digestive contents for further genetic analysis. We deposited all animals in the collections of the Technical University of the Atlantic, São Vicente, the future Natural History Museum of Cabo Verde.

### 2.3 DNA extraction and sequencing

We divided the digestive tracts into three sections (stomach—S, small intestine—SI, and large intestine—LI). We performed the DNA extraction of the digestive contents of 30 individuals per island, in a total of 90 specimens, for each section separately, including blanks, using the Stool DNA Isolation Kit (Norgen Biotek Corp., Canada), following the manufacturer's instructions.

We amplified the DNA of the three sections with two genetic markers already validated in previous studies (Pinho et al., 2018). For invertebrates we used a modified version of the IN16STK‐1F/IN16STK‐1R primers, targeting the mitochondrial 16S rRNA (Kartzinel & Pringle, 2015; Pinho et al., 2018), and for plants, we used the g/h primers targeting the short P6‐loop of chloroplast trnL (UAA) (Taberlet et al., 2007). We performed all PCRs, including blanks, as described in Pinho et al. (2018). Then, we prepared libraries following the Illumina MiSeq protocol “16S Metagenomic Sequencing Library Preparation” (https://support.illumina.com). Finally, we sequenced the samples in the MiSeq sequencer (Illumina) using the MiSeq Reagent Kit V2 (Illumina, San Diego, CA, USA) for an expected average of 17,000 paired‐end reads per sample.

We processed the obtained DNA sequences at the bioinformatics level using tools incorporated in the software package OBITtools (http://metabarcoding.org/obitools), which include the alignment of the sequences obtained and the filtering of sequencing errors, to obtain the molecular operational taxonomic units, MOTUs (Pinho et al., 2018). In the final dataset, we removed all the samples with less than 500 reads and within kept samples, we also excluded haplotypes representing less than 1% of the total number of reads of that sample (Mata et al., 2016). Finally, to taxonomically identify the MOTUs present in the mice diet, we compared the obtained haplotypes with our reference database and with those available in the GenBank database (https://www.ncbi.nlm.nih.gov/genbank/). We classified sequences that had less than 90% of similarity with known species only to the class level, the ones with values between 90% to 95% to the family level, and the remaining sequences with similarity values above 95% to the genus or species level. We considered only species or genera known to occur on our sampled sites, or the surrounding islands of the archipelago. When a haplotype matched more than one species or genus, then a higher ranking would be attributed (e.g., family). If more than one haplotype corresponds to the same taxon, we attributed a number to each. We removed the haplotypes identified as contaminations (e.g., mice, bait, or human DNA).

### 2.4 Data analysis

We estimated the frequencies of occurrence (FO) of plants and invertebrates in samples for the three sections. Using R software version 4.0.2, we performed a permutational multivariate analysis of variance (PERMANOVA) using the vegan package (ADONIS function) to compare the composition of the contents of all sections (https://CRAN.R‐project.org/package=vegan). For the same purpose, we performed a pairwise multilevel comparison (parwise.adonis function). We also carried out a homogeneity of dispersion test (PERMDISP) to ensure the importance of the PERMANOVA test, as it presumes an equal dispersion of values among groups. Additionally, we performed a similarity percentage analysis, using the vegan package (simper function), to infer the contribution of each prey to the differentiation among sections.

Lastly, we classified the impact of mice on the taxonomically identified diet items into three categories: negative, positive, or non‐identified. We consider it as negative if the items were native or endemic plants/invertebrates, plants of economic importance (e.g., for human or domestic animals' consumption), or invertebrates with essential ecological functions, such as pollinators. We considered it as a putative positive if the items were exotic plants/invertebrates and as non‐identified if the items were identified with a low taxonomic resolution or if the impact could not be classified. We compared the FO differences of each category between prey groups (plants vs. invertebrates), gastrointestinal sections (stomach vs. intestines) and islands using chi‐square tests.

## 3 RESULTS

After data filtering, we obtained 20,000 reads per sample on average. We were able to identify 131 diet items of eight taxonomic classes in the 90 mice from the three islands, 73 of which corresponded to plants and 58 to invertebrates (Table A1). Plants were distributed among three classes, 20 orders, and 30 families, with Poaceae and Fabaceae as most frequent overall. We identified invertebrates from five classes, 15 orders, and 31 families, with Apidae and Aphididae as the most frequent families. Both plant and invertebrate items were present in similar frequencies in the overall diet (63% and 72%, respectively). Several taxa were present exclusively in one section, in total 54% of plants items were only in the intestines (e.g., Arecales and Apiales orders), and 65% of invertebrate items were only in the stomach (e.g., Araneae and Mesostigmata orders). Fifty MOTUs had FOs below 1% and were only present in one sample.

**FIGURE 2:**
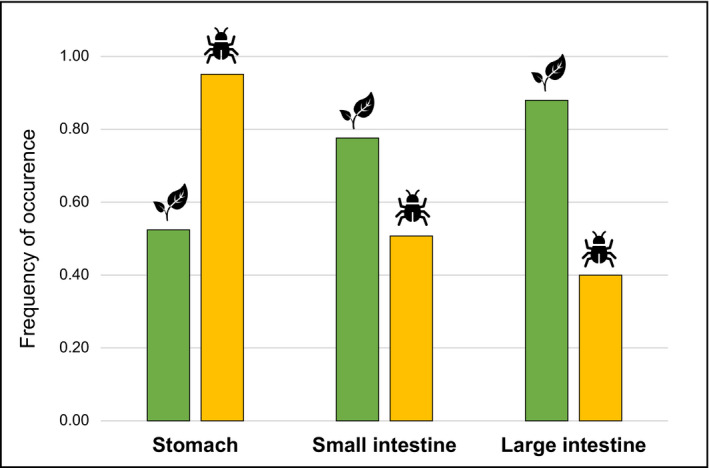
Metabarcoding results per gastrointestinal section. Frequencies of occurrence of plants (in green) and invertebrates (in yellow) in the *Mus musculus* stomach and intestines (small and large) samples

Regarding the gastrointestinal track sections analysed, we obtained significant differences in the diet composition of the stomach in comparison to the two intestinal sections (S vs. SI: *F* = 3.46, *p* = .003; S vs. LI: *F* = 7.68, *p* = .003; SI vs. LI: *F* = 1.65, *p* = .120) and no effect of data dispersion on the results. Frequencies of plants and invertebrates were similar in the small and large intestines (Figure 2), yet both sections' estimates were significantly different from the stomach ones (plants – S vs. SI: *p* = .001; S vs. LI: *p* = .001; SI vs. LI: *p* = 1.000; invertebrates – S vs. SI: *p* = .008; S vs. LI: *p* = .001; SI vs. LI: *p* = .062). These differences were observed at the MOTU, family, and order levels. Additionally, we observed a higher frequency and diversity of invertebrates in the stomach and of plants in the intestines (Figure 2).

**FIGURE 3:**
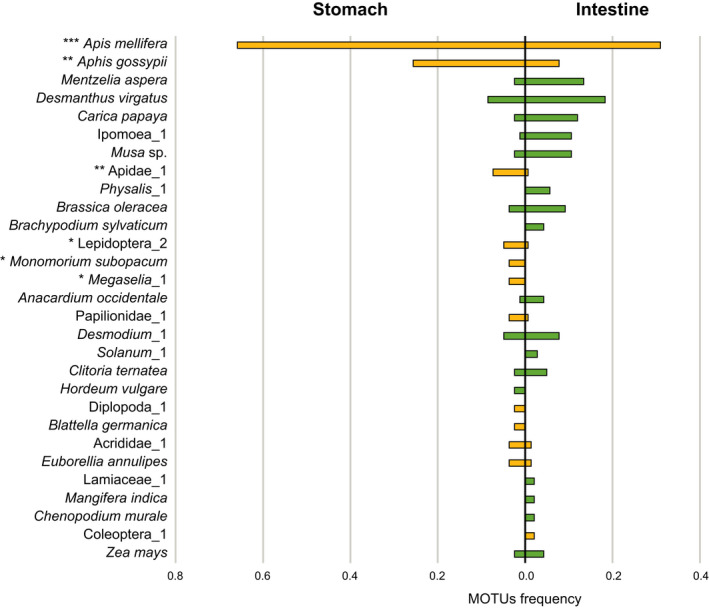
Results of the similarity percentage analysis. Frequency of occurrence of 30 Molecular Operational Taxonomic Units (MOTUs) with the highest contribution to differences between the stomach and intestine of *Mus musculus*. Plant MOTUs are represented in green and invertebrates in yellow. Magnitude of significance levels shown with asterisks: ***p < .001; **p < .01; *p < .05

The similarity percentage analysis revealed that six MOTUs, five families, and seven orders contributed significantly to differences between sections (Figure 3; Table A1). The MOTUs that contributed significantly to the differences between sections were all invertebrates. In particular, *Apis mellifera* Linnaeus, 1758 and *Aphis gossypii* Glover, 1877 had a high contribution and were also the most frequent preys overall. The bee and aphid species were present in 66% and 26% of the stomachs, respectively, whereas in the intestines the FOs were only 31% and 8%, respectively. Other three introduced plant species, *Mentzelia aspera* L., *Desmanthus virgatus* (L.) Wild., and *Carica papaya* L. had differences in the FO between sections greater than 10%, all presenting higher frequency in the intestines (Figure 3).

**FIGURE A1:**
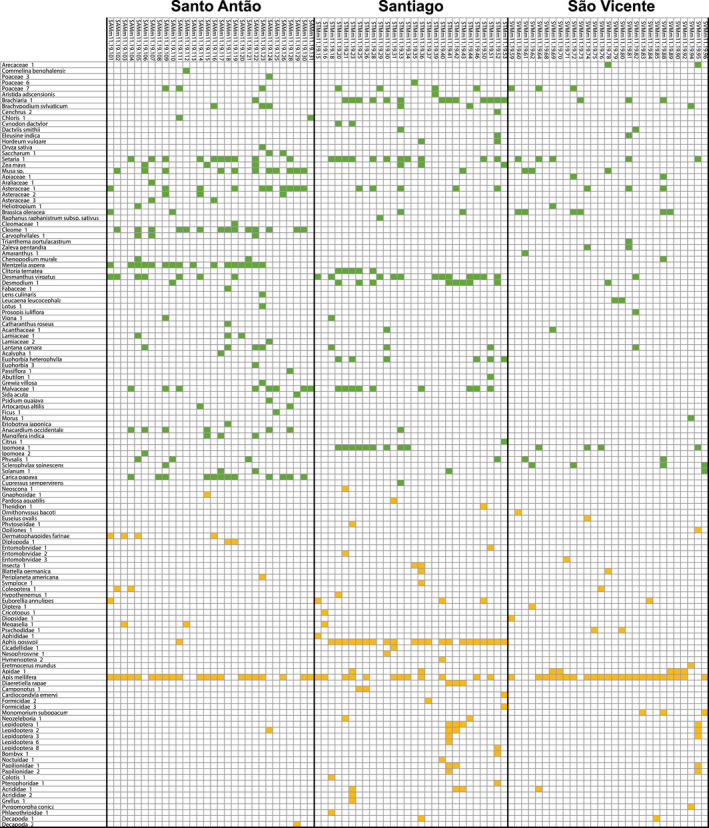
Heatmap representing the distribution of the different diet items in the *Mus musculus* samples of the three islands (Santo Antão, Santiago and São Vicente). Occurrences of plant items are represented in green and of invertebrate items in yellow

Regarding the impact of this rodent, we identified with a high taxonomic resolution 79 items (27 invertebrates and 52 plants), although the reference collection of DNA sequences for Cabo Verde, mostly for invertebrates, is still incomplete. Therefore, we verified that the impact of this invader in the ecosystem is predominantly negative, as at least 49/50% (unbalanced/balanced per FO, respectively) of the ingested items were native, endemic, or economically important taxa, and only 12/19% (unbalanced/balanced per FO, respectively) of the items were exotics (Table A1). The impact pattern was similar across the three sampled islands (*χ* ^2^ = 1.87, *p* = .761; Figure A1) and gut sections (*χ* ^2^ = 0.66, *p* = .719). Impact patterns were significantly different between invertebrates and plants (*χ* ^2^ = 8.95, *p* = .011), with a higher negative impact on plants. The economically important plants included corn *Zea mays* L., banana *Musa* sp., cabbage *Brassica oleracea* L., radish *Raphanus raphanistrum* subsp. *sativus* (L.) Domin., breadfruit *Artocarpus altilis* (Parkinson ex F.A. Zorn) Fosberg, mango *Mangifera indica* L., papaya *Carica papaya* L., and plants of the genus *Solanum* that includes potatoes, tomatoes, and eggplant, among other vegetables (Table A1). Mice also consumed native and endemic invertebrate species including spiders (e.g., *Pardosa aquatilis* Schmidt & Krause 1995), ants (e.g., *Monomorium subopacum* (Smith 1858)) and European honeybees *Apis mellifera*. The data show that they also consumed invasive plants, such as *Lantana camara* L. and Mexican fireplant *Euphorbia heterophylla* L., along with invertebrate pests, such as cockroaches (e.g., *Blattella germanica* (Linnaeus 1767) and *Periplaneta americana* (Linnaeus 1758)) and the cotton aphid *Aphis gossypii* Glover 1877.

## 4 DISCUSSION

To our knowledge, this is the first study to explore diet estimate differences along the gastrointestinal tract using DNA‐metabarcoding techniques. In this work, we present the first DNA‐based data on the diet and impact of *Mus musculus*, using Cabo Verde as a model. Our results show that the house mouse consumed a wide range of prey items in the sampled islands, as the FOs of most items were low. This confirms the generalist diet and opportunistic feeding behavior of this species as observed in other islands (Angel et al., 2009; Le Roux et al., 2002). We were able to observe that the frequency of plants and invertebrates were similar overall; however, plants had a slightly higher incidence. This was expected since we actively sampled individuals in crop areas. Nevertheless, studies that investigated the seasonal variation of the diet of *M*. *musculus* in other islands showed a similar pattern (Copson, 1986; Le Roux et al., 2002).

The analyses of different sections of the gastrointestinal tract provided different views of the house mouse diet. Although no significant differences were detected between the two sections of the intestines, disparities between the stomach and the intestines were evident. We observed a higher frequency and diversity of invertebrates in the stomach and plants in the intestines. This may be primarily due to differences in the cellular structures between plants and animals, particularly in the cell walls and fibrous tissues. Plant items have very thick cell walls arranged in complex networks predominantly composed of cellulose, hemicellulose, and pectic polysaccharides (Jarvis, 2011; McDougall et al., 1996). This composition makes plant items harder to break down and consequently more resistant to enzymes of the gastrointestinal tract (Holland et al., 2020). This leads to harder digestion of plants, in comparison with animal items, and subsequently slower absorption along the gastrointestinal tract (Tomé, 2013). Since mice consume raw plants, the DNA is protected by the cell wall and remains poorly detected in the stomach. The subsequent gastrointestinal compartments take a much important part in their digestion (Rizzi et al., 2012; Tomé, 2013; Wilcks et al., 2004). Previous studies showed that plant tissues were most effectively digested in the small intestine of rodents, due to the action of pancreatic enzymes (Wilcks et al., 2004). As shown in our results, one is more likely to detect DNA from plants in the intestines rather than the stomach. Moreover, depending on the type, section, and maturation of the plant, the fiber concentration will vary, leading to distinct digestibility rates (Albrecht et al., 1987; Buxton et al., 1995). For instance, grasses usually present more fiber than legumes, especially in the leaves, making them harder to digest and to be absorbed in the gut (Buxton & Redfearn, 1997). In our study, Poaceae was the most frequent plant family and one of the families that contributed to the differences between stomach and intestines. This is probably due to its higher fiber concentration which presumably allowed Poaceae plants to persist longer time in the gastrointestinal tract, therefore having a higher incidence in the intestines. Moreover, when we extracted plant DNA of the intestines, we were also extracting DNA of the faeces within it. Since the DNA detectability half‐life is twice as high in faeces compared to stomachs (Egeter et al., 2015), the faeces content in the intestines will also contribute to a higher DNA detectability of plants in that section.

Comparatively, animal DNA will be already further degraded when it reaches the lower sections of the gastrointestinal tract (Tomé, 2013), compromising its detectability. This was probably the reason why we were able to detect higher frequencies of invertebrates in the stomach than in the intestines. Additionally, the most frequent invertebrates consumed by mice, and the ones that contributed the most to differences between sections, seem to be soft‐bodied (e.g., *Apis mellifera* and *Aphis gossypii*). These items are probably more easily digested and are already mostly absorbed when they reach the intestines, leading to a lower detection of its DNA in the lower parts of the gastrointestinal tract. Even though metabarcoding has the extraordinary capacity to detect small, soft, and invisible items (Pompanon et al., 2012), based on our results, the detection of these preys will probably be more efficient in stomach contents rather than in the intestines. Therefore, we advise studies targeting the detection of soft‐bodied invertebrate preys to use preferentially stomach samples and those targeting plant or harder invertebrate items to use preferentially intestine samples. On the other hand, extraction methods can be adjusted to account for lower digestion of prey. For instance, if only the stomach is available, we advise using specific extraction and amplification methods targeting plants.

In this work, we verified that the general environmental impact of mice in Cabo Verde is predominantly negative similarly to other island ecosystems (Angel et al., 2009). Studies using traditional methods of stomach content analysis on Southern Ocean islands, as the Guillou and Macquarie Islands, similarly showed that mice consume a wide range of native taxa (Copson, 1986; Le Roux et al., 2002). We showed that this invader consumes several plants with human and economic importance. Corn production is one of the most important crops in Cabo Verde, with around 6,000 tons per year (MAA, 2021). We observed that 10% of the individuals ingested corn, which coupled with high population densities could imply significant losses in production, considering that 1 ton of corn is equivalent to 108.100 Cabo Verdean escudos, or 1.190 dollars (I. Gomes pers. comm.). Also summing the losses in other crops, this invader poses a major threat to the national economy of this archipelago. Similarly, in Tanzania and Australia, mice can cause losses of up to 15% of the national agricultural production, equivalent to 40–45 million dollars of losses in the worst seasons (Singleton, 1997). In our results, we were also able to detect that this invader feeds on at least nine native plant species, as *Eleusine indica* (L.) Gaertn and *Sida acuta* Burm. fil. (Table A1). Metabarcoding does not allow us to identify the section of the plant that is consumed; however, this invader can be a great threat for the native flora if it is consuming seeds. In Marion Island, in the sub‐Antarctic Indian Ocean, mice almost extirpated the native sedge *Uncinia compacta* R. Br. due to seed predation (Smith & Steenkamp, 1990). The impact on invertebrates is also considerable since these rodents consume several native and endemic species, most of them with vital ecological functions. This is the case of the European bee, a species in decline worldwide, that plays an active role in the pollination of several plants, including crops such as cabbage and beans (Themudo et al., 2020). In Antipodes Island, located south of New Zealand, mice were considered accountable for the local extirpation of several invertebrate species (Marris, 2000). Likewise, on Marion Island, they are responsible for the absence of the flightless moth *Pringleophaga kerguelensis* Enderlein 1905 (Vári, 1971).

Mice can also present a putative positive impact by consuming some introduced and invasive herbaceous, as is the case in Cabo Verde, for example, *Lantana camara* and *Euphorbia heterophylla*, two of the main invasive plants in several natural and agricultural tropical regions (Romeiras et al., 2016). The latter is resistant to some herbicides, thus it needs to be manually removed before farming (Wilson, 1981). Mice can also play a role in domestic pest control, such as cockroaches (e.g., *Blattella germanica* and *Periplaneta americana*) and the cotton aphid *Aphis gossypii*, even though some may be resultant of secondary consumption of host plants (Mata et al., 2016). The latter is an important agricultural pest since it can have several hosts and transmit several viruses important to crops ( Dedryver et al., 2010). Several crops grown in Cabo Verde can be hosts for this aphid, such as papaya, cashew, breadfruit, and banana, among others (Dedryver et al., 2010).

Optimized measures are needed to control, monitor and eradicate invasive rodents (Stenseth et al., 2003). These measures imply great expenses with equipment and human resources; therefore they must be carried out accurately to avoid resource waste (Mwebaze et al., 2010). The results of Stenseth et al. (2003) suggest that government‐level funding for rodent pest control should be devoted to research instead, especially in developing countries. These authors go even further, stating that not doing so can cost dearly in terms of lost income and food supplies for people. Consequently, additional research is crucial to infer the relative importance of the impacts of this rodent in the Cabo Verdean agriculture and economy, and if eradication is the best solution. If so, there is a need to deliberate on potential surrogates for the positive role of mice. For instance, in this study, we did not consider the impact that this rodent can have on vertebrates. However, we know that in Cabo Verde, mammal invaders already contributed to the extinction of endemic species (Mateo et al., 2005; Medina et al., 2021), therefore, it is important in the future to access the impact that *M*. *musculus* has on seabirds and terrestrial and marine reptiles. Further studies on other islands, particularly in Seychelles, show that the prevention, eradication, and control of rodents would very positively affect government revenues even after discounting the costs of such actions (Mwebaze et al., 2010). An eradication plan of invasive mammals is already taking place in Cabo Verde on Santa Luzia Island (Alho et al., 2022). This is a particularly promising plan due to the reduced area of the island since in another small island of the Macaronesian region mice were already successfully eradicated (Olivera et al., 2010). On populated islands with a strong agriculture presence, as is the case of this study, the likelihood of eradication failure can be high (Holmes et al., 2015); however, these islands could highly benefit from a rodent control program to reduce the impact of mice on agriculture.

In conclusion, our results showed the need to analyse only two gastrointestinal tract sections (stomach and large intestine) to obtain robust diet representations, increasing the cost‐effectiveness of DNA‐metabarcoding approaches. In general, the use of this DNA‐based approach enabled the identification of a wide variety of mice diet items in a much faster and simplified way compared to other traditional approaches, as shown in previous studies (Gil et al., 2020). Additionally, by uncovering which native prey species are most frequently predated by this invader, this approach allowed to evaluate efficiently which ones are at the highest risk. Thus, our framework can be an asset for studies on the impact of invasive species on other islands or threatened areas.

## CONFLICT OF INTEREST

The authors declare that they have no competing interests.

## AUTHOR CONTRIBUTIONS

**Catarina J. Pinho:** Formal analysis (lead); Investigation (equal); Visualization (lead); Writing – original draft (lead); Writing – review & editing (equal). **Evandro P. Lopes:** Funding acquisition (supporting); Investigation (supporting); Resources (supporting); Writing – review & editing (equal). **Joana Paupério:** Conceptualization (supporting); Formal analysis (supporting); Funding acquisition (supporting); Methodology (equal); Resources (supporting); Validation (equal); Writing – review & editing (equal). **Isildo Gomes:** Funding acquisition (supporting); Investigation (supporting); Resources (supporting); Writing – review & editing (equal). **Maria M. Romeiras:** Data curation (supporting); Funding acquisition (supporting); Methodology (supporting); Resources (supporting); Writing – review & editing (equal). **Raquel Vasconcelos:** Conceptualization (lead); Data curation (lead); Formal analysis (supporting); Funding acquisition (lead); Investigation (equal); Methodology (equal); Project administration (lead); Supervision (lead); Writing – original draft (equal); Writing – review & editing (equal).

## Data Availability

The datasets supporting this article have been uploaded as part of Appendix A and in the GenBank database (PRJNA801221 accession code; https://www.ncbi.nlm.nih.gov/sra/PRJNA801221).

## APPENDIX A

**TABLE A1:** Taxonomic identifications, haplotype sequences, and frequency of occurrence (FO) of the MOTUs present in the diet of *Mus musculus*

Phyllum	Class	Order	Family	Final_ID	DNA sequences	ST	SI	LI	Total	Impact
Tracheophyta	Liliopsida	Arecales	Arecaceae	Arecaceae_1	atctttattttgagaaaacaagggtttataaaactagaataaaaaaag	0.000	0.004	0.004	0.009	−
		Commelinales	Commelinaceae	*Commelina benghalensis*	atccttaagatttttaaaactagaaaaaagg	0.000	0.000	0.004	0.004	+
		Poales	Poaceae	*Aristida adscensionis*	atctcttttttgaaaaacatgtggttctaaaactagaacccaaaggaaaag	0.000	0.000	0.004	0.004	−
				*Brachypodium sylvaticum*	atccgtgttttgagaaaacaaggaggttctcgaactaaaatacaaaggaaaag	0.000	0.000	0.004	0.004	+
				*Cenchrus ciliaris*	atcccttttttgaaaaacaagtggttctaaaactagaacccaaaggaaaag	0.000	0.004	0.000	0.004	−
				*Chloris_1*	atccctgttttgaaaaacaagtggttctcaaactagaacccaaaggaaaag	0.000	0.000	0.009	0.009	−
				*Cynodon dactylon*	atcccttttttgaaaaacaagtggttctcaaaccagaacccaaaggaaaag	0.004	0.000	0.004	0.009	+
				*Dactylis smithii*	atccgtgttttgagaaaacaaagaaaggggttctcgaactagaatacaaaggaaaag	0.000	0.004	0.004	0.009	−
				*Eleusine indica*	atccctttttttcattttaaaaacaagtggttctcaaactagaacccaaaggaaaag	0.004	0.000	0.009	0.013	−
				*Hordeum vulgare*	atccgtgttttgagagggggattctcgaactagaatacaaaggaaaag	0.009	0.000	0.000	0.009	−
				*Oryza sativa*	atccatgttttgagaaaacaagcggttctcgaactagaacccaaaggaaaag	0.004	0.000	0.000	0.004	−
				Poaceae_3	atccctttttttaaaaacaagtggttctcaaactggaacccaaaggaaaag	0.000	0.004	0.000	0.004	?
				Poaceae_6	atccgtgttttgagaggggggttctcaaactagaatacaaaggaaaag	0.000	0.000	0.004	0.004	?
				Poaceae_7	atccgtgttttgagaaaacaaggggttctcgaactagaatacaaaggaaaag	0.031	0.022	0.000	0.054	?
				*Saccharum*_1	atccccttttttgaaaaaacaagtggttctcaaactagaacccaaaggaaaag	0.000	0.000	0.009	0.009	−
				*Setaria*_1	atcccttttttgaaaaaacaagtggttctcaaactagaacccaaaggaaaag	0.054	0.045	0.045	0.143	?
				*Urochloa*_1	atccctcttttgaaaaaacaagtggttctcaaactagaacccaaaggaaaag	0.036	0.022	0.045	0.103	−
				*Zea mays*	atcccttttttgaaaaacaagtggttctcaaactagaacccaaaggaaaag	0.009	0.022	0.004	0.036	−
		Zingiberales	Musaceae	*Musa* sp.	atccttattttgagaaaacaaaggtttataaaactagaatttaaaag	0.009	0.018	0.049	0.076	−
	Magnoliopsida	Apiales	Apiaceae	Apiaceae_1	atcctattttccaaaaacaaacaaaggcccagaaggtgaaaaaag	0.000	0.009	0.000	0.009	−
			Araliaceae	Araliaceae_1	atcctgttttccgaaaacaaacaaaggttcagaaggcgaaaaaagg	0.000	0.000	0.004	0.004	?
		Asterales	Asteraceae	Asteraceae_1	atcacgttttccgaaaacaaacaaaggttcagaaagcgaaaataaaaaaag	0.004	0.000	0.009	0.013	−
				Asteraceae_2	atcacgttttccgaaaacaaataaaggttcagaaagcgaaaataaaaaag	0.000	0.000	0.013	0.013	?
				Asteraceae_3	atcacgttttccgaaaacaaacaacggttcagaaagcgaaaatcaaaaag	0.000	0.004	0.004	0.009	?
		Boraginales	Heliotropiaceae	*Heliotropium*_1	atcctgttttccgaaaacaaagaaaggttcagaaagcaaaaaaag	0.000	0.009	0.000	0.009	−
		Brassicales	Brassicaceae	*Brassica oleracea*	atcctgggttacgcgaacaaaacagagtttagaaagcga	0.004	0.000	0.000	0.004	−
				*Raphanus raphanistrum* subsp. *sativus*	atcctgagttacgcgaacaaaccagagtttagaaagcgg	0.000	0.004	0.000	0.004	−
			Caricaceae	*Carica papaya*	atcctgttttacgagaacaaacaaaacaagagttcagaaagcgaaaaaagggg	0.000	0.004	0.000	0.004	−
			Cleomaceae	Cleomaceae_1	atcctggtttccgcgaacaaacaagagtttagaaagcgagaaaaaagg	0.004	0.000	0.000	0.004	?
				*Cleome*_1	atcctggtttacgcgaacaaacaagagtttagaaagcgagaaaaaagg	0.031	0.027	0.031	0.089	+
		Caryophyllales	NI	Caryophyllales_1	ctcctttttcaaatcaaaagaaaaaaaaaataaagattcataaagcaagaaaaaag	0.009	0.000	0.004	0.013	?
			Aizoaceae	*Trianthema portulacastrum*	ctcctttttttttcaaaagcaaaaaacaaaaaataaggattcagaaagcaagaaaaag	0.004	0.004	0.004	0.013	+
				*Zaleya pentandra*	ctcctttttttcaaaagcaaaaaaataaggattcagaaagcaagaaaaaag	0.004	0.004	0.004	0.013	−
			Amaranthaceae	*Amaranthus*_1	ctcctttttcaaaagtaaaaaaaaaaaatacggattcagaaagcaagaataaaaaaaag	0.004	0.000	0.000	0.004	−
				*Chenopodium murale*	ctccttttgtcaaaagcaaaaaatactcaaaagaaaaaaataataaaaaagcaagaaaaaaaaag	0.000	0.004	0.009	0.013	+
		Cornales	Loasaceae	*Mentzelia aspera*	atcctgttttccgaaaacaaacaaaggttcagaaagcgaaaataaaaaaag	0.009	0.040	0.045	0.094	+
		Fabales	Fabaceae	*Clitoria ternatea*	atcctcttttccaaaattttccaaaaacaaaaaacttaagaaagtgaaaaaagag	0.009	0.022	0.009	0.040	−
				*Desmanthus virgatus*	atcctgttttccgaaaaccaagaagagttcagaaagggagaatcaaaataaaaaaa	0.000	0.000	0.004	0.004	−
				*Desmodium*_1	atccttttttccgtaaacgaagaaaagttcagaaaagaaaggtataatcaaaaaaaag	0.000	0.000	0.004	0.004	−
				Fabaceae_1	atcctgttttccgaaaacaaagaacaaagaaaaagaagagtttagaaagcgagaataaaaaatcaaag	0.000	0.000	0.004	0.004	?
				*Lens culinaris*	atccttctttccgaaaacaaatcaaagttcagaaagtgaaaatcaaaaaag	0.004	0.000	0.000	0.004	−
				*Leucaena leucocephala*	atcctgttttccgaaaagcaagaagagttcagaaagggagaatcaaaataaaaaaag	0.004	0.004	0.009	0.018	−
				*Lotus*_1	atcctgctttacgaaaacaagggaaagttcagttaagaaagcgacgagaaaaaagg	0.000	0.000	0.004	0.004	−
				*Prosopis juliflora*	atcctgttttcggaaaaccaagaagagttcagaaagggagaataaaaaaag	0.000	0.000	0.004	0.004	?
				*Vigna*_1	atcctgttttctgaaaacaaagaaaaattcagaaagttataataaaaaagg	0.000	0.000	0.009	0.009	−
		Gentianales	Apocynaceae	*Catharanthus roseus*	atccagttttccacaaacacaaacaaaggttcagaaaacgaaaaagg	0.004	0.000	0.000	0.004	−
		Lamiales	Acanthaceae	Acanthaceae_1	atcctcttttcgcaagacaaaggttcagaaaacgaaaaagg	0.000	0.000	0.009	0.009	?
			Lamiaceae	Lamiaceae_1	atcctgttttctcaaaacaaaggttcaaaaaacgaaaaaagg	0.000	0.009	0.004	0.013	?
				Lamiaceae_2	atcctgttttctcaaaacaaaggtttaaaaaacgaaaaaaaaaaag	0.000	0.000	0.004	0.004	?
			Verbenaceae	*Lantana camara*	atcccgttttctcaaaacaaaggttcagaaaacgccaaaggcg	0.000	0.009	0.000	0.009	+
		Malpighiales	Euphorbiaceae	*Acalypha*_1	atcctgttttccgaaaacaaaaaaaggttcataaagacagaataaaaaagg	0.000	0.004	0.000	0.004	+
				*Euphorbia heterophylla*	atcctgttttccgaaaacaaaaaaagtttcataaagacagaaaaaaaaaaaaaaaaagaag	0.013	0.000	0.013	0.027	+
				*Euphorbia*_3	atcctgttttccgaaaacagaaaaagaaaagtttcataaaaacagaaaaaaacaaggaag	0.000	0.004	0.000	0.004	?
			Passifloraceae	*Passiflora*_1	atcctgtttttcgaaaacaaaaaaacaacaaaggttcataaagacagaatcagaataaataaag	0.000	0.000	0.004	0.004	?
		Malvales	Malvaceae	*Abutilon*_1	atcctattattttacgaaaataaacataaacaaaggttcagcaagcgaaaataataaaaaaggaaag	0.000	0.000	0.004	0.004	?
				*Grewia villosa*	atcctattattttacgaaaataaacataaacaaaggttcagcaagcgagaataaaataaaataataaaaaaag	0.004	0.000	0.000	0.004	−
				Malvaceae_1	atcctattattttacgaaaataaacataaacaaaggttcagcaagcgagaataataaaaaaggaaag	0.013	0.022	0.063	0.098	?
				*Sida acuta*	atcctattattttacgaaaataaacagaaacaaaggtttagcaagcgagaataataataaaaaaggaaag	0.000	0.000	0.004	0.004	−
		Myrtales	Myrtaceae	*Psidium guajava*	atcctgttttacgaaaaccaacaaaacaataagggttcataaagcgagaataaaaaaaggatag	0.000	0.000	0.004	0.004	−
		Rosales	Moraceae	*Artocarpus altilis*	atccggttttctgaacccttgtttgttttcagaaagcgataataaaaaag	0.004	0.004	0.009	0.018	−
				*Ficus*_1	atccggttttctgaaaacaaacaagggttcagaaggcgataataaaaaag	0.000	0.000	0.004	0.004	−
				*Morus*_1	atccggttttctgaaaacaaacaagggttcagaaagcgataatacaaaag	0.000	0.000	0.004	0.004	?
			Rosaceae	*Eriobotrya japonica*	atcctgttttatgaaaataaacaagggtttcataaaccgaaaataaaaaag	0.000	0.000	0.004	0.004	−
		Sapindales	Anacardiaceae	*Anacardium occidentale*	atcctattttacgagaacaaaaacaaacagggggtcagaacgggagaaaaaaaag	0.004	0.009	0.018	0.031	−
				*Mangifera indica*	atcctattttacgagaacaaaaacaaacaaggggtcagaacgggaaaaaaaaag	0.000	0.004	0.000	0.004	−
			Rutaceae	*Citrus*_1	atcctcttctcttttccaagaacaaacaggggttcagaaagcgaaaaagggg	0.000	0.004	0.000	0.004	−
		Solanales	Convolvulaceae	*Ipomoea*_1	atcctgttttccgaaaacaaacaaaagttcagaaaaaaag	0.004	0.004	0.013	0.022	−
				*Ipomoea*_2	atcctgttttccgaaaacaaacaaaggttcagaaaaaaag	0.000	0.000	0.004	0.004	−
			Solanaceae	*Physalis*_1	atcctgttttctgaaaacaaacaaaggttcagaaaaaaag	0.000	0.013	0.022	0.036	−
				*Sclerophylax spinescens*	atcctgttttctgaaaacaaacaaaggttcagcaaaaaag	0.004	0.004	0.013	0.022	+
				*Solanum*_1	atcctgttttctcaaaacaaacaaaggttcagaaaaaaag	0.000	0.000	0.009	0.009	−
	Pinopsida	Pinales	Cupressaceae	*Cupressus sempervirens*	atccgatttctagaaacaataggttcctttccgagaacgg	0.000	0.000	0.004	0.004	?
Arthropoda	Arachnida	Araneae*	Araneidae	*Neoscona*_1	tgatttaaaggtcgaacagacctacttttaaaattgttgcatctataaagttacattaattcaacatcgaggtcgcaatcatttttttaaataagaactttagaaaaata	0.004	0.000	0.000	0.004	−
			Gnaphosidae	Gnaphosidae_1	atttttataagtcgaacagactttatatattcaatcttgcttgaataggataattaattcaacatcgaggtcgtaaacatttatttttattaggactttaaattaata	0.004	0.000	0.000	0.004	−
			Lycosidae	*Pardosa aquatilis*	ttttttaaaagtcgaacagacttcctttattaactattgcgtaaattaggaaaaattaaatcaacatcgaggtcgcaatctttattttaaataagatcttataaattata	0.004	0.000	0.000	0.004	−
			Theridiidae	*Theridion*_1	tatattaaaggtcgaacagacctacttatataactactgcgttattatagaataataattcaacatcgaggtcataaacattatttttaataagagctttaaaataata	0.004	0.000	0.000	0.004	−
		Mesostigmata*	Macronyssidae	*Ornithonyssus bacoti*	tgatttaaaagatgaacaatctttcttatatagtctctccaccatatagacacattaattcaacatcgaggtcgcaaactactatatcaatatgaactctccatagtaa	0.004	0.000	0.000	0.004	?
			Phytoseiidae	*Euseius ovalis*	caatttaaaagatgaacaatctcccttagtaattctctacaacaactaggtgtgtcaaattcaacatcgaagtcgcaaactactataccaatatgaactctccatagtaa	0.004	0.000	0.000	0.004	?
				Phytoseiidae_1	aaatttaaaagatgaacaatctttctattttaatattcttctattaatagatatcttaattcaacatcgaggtcgtaaactattataccaatatgttctatccataataa	0.004	0.000	0.000	0.004	?
		Opiliones	NI	Opiliones_1	aaaattaatagtcgaacagactaactcatatctatatttctagaaacaatgtttttaattcaacatcgaggtcgcaatcttactcactaataagaactctaaaagtaaa	0.004	0.000	0.000	0.004	?
		Sarcoptiformes	Pyroglyphidae	*Dermatophagoides farinae*	aaaatcatcagacgaacagtctgactaactctagccccttcgccaagtagtggttttaatccaacatcgaggtcccaaacccccttaaagataagaactcataaagggta	0.004	0.004	0.009	0.018	?
	Diplopoda	NI	NI	Diplopoda_1	gaatttaaaggtcgaacagaccttctttaacaactactgcaccaataagacttcttattccaacatcgaggtcgcaaacattttcgtcaatatgaactctcaaaaaata	0.009	0.000	0.000	0.009	?
	Entognatha	Collembola	Entomobryidae	Entomobryidae_1	aaatttaatagtcgaacagactttcttataaaaatactgctcttttaagaaattttaattcaacatcgaggtcgcaaaaaattttgtcgataagaactctaaaaaatca	0.000	0.000	0.004	0.004	−
				Entomobryidae_2	aaatttaatggtcgaacagaccatttaatattaactactgcactaaaaaaactttttaattcaacatcgaggtcgcaaacacatctattaataagaactctcaagatata	0.004	0.000	0.000	0.004	−
				Entomobryidae_3	atattttatagacgaacagtctagcttttaaaatttctgcactttaaagcgtatttaattcaacatcgaggtcgcaaactaaaatgtcgataagaactctcaattttaa	0.000	0.004	0.000	0.004	−
	Insecta	NI*	NI	Insecta_1	gaatttaatagtcgaacagactaactcaaaagaaaaactaccccccaaaattatcttaattcaacatcgaggtcgcaaacttaactataaataagaactctccagtaaaa	0.009	0.000	0.000	0.009	?
		Blattodea*	Blattidae*	*Blattella germanica*	gattttaaaggtcgaacagacctaacatacttaaacttctacacctaatgtttatcttaatccaacatcgaggtcgcaaccctttttgtcgataagaactcttaaaaaaga	0.009	0.000	0.000	0.009	+
				*Periplaneta americana*	gattttaaaggtcgaacagacctaaacaaattgaaattctacacccaaattaaatcttaatccaacatcgaggtcgcaaccccatttatcaataagaactctcaaaaaaga	0.004	0.000	0.000	0.004	+
				*Symploce*_1	gattttaaaggtcgaacagacctaacattattaaacttctacacctaatgtttatcttaatccaacatcgaggtcgcaaccctttttgtcgatattaactctcaaaaagga	0.004	0.000	0.000	0.004	−
		Coleoptera	NI	Coleoptera_1	aaaattaaaagacgaacagtcctacttctccagccccttcgccaaagagtaattttaatccaacatcgaggtcccaaacccccctaaagataagaactcacaagaagga	0.000	0.000	0.013	0.013	?
			Curculionidae	*Hypothenemus*_1	aatttcaaaagtcgaacagactcaatttcccagcttctacaccaagaatttattttaatccaacatcgaggtcgcaatctcttctttcgataagaactctcaagaaaaa	0.000	0.004	0.000	0.004	−
		Dermaptera	Anisolabididae	*Euborellia annulipes*	aattttaatagtcgaacagactaaaattataaactcctgcatttatattttattttaatccaacatcgaggtcgcaatctctcttgttaataagttctctctaagagaa	0.013	0.009	0.000	0.022	+
		Diptera*	NI	Diptera_1	gattttaatagtcgaacagactaaattttcaagcttctgcacctaaaatttatcttaatccaacatcgaggtcgcaatcctctataataataagaactcaaaaaagaga	0.004	0.000	0.000	0.004	?
			Chironomidae	*Cricotopus*_1	gaaaatataagtcgaacagactttgaatttaaacttctacacctaaattaattcttagtccaacatcgaggtcgcaatcttttttatcgatttgaactctccaaaaaaa	0.004	0.000	0.000	0.004	?
			Diopsidae	Diopsidae_1	gatttaaaaagtcgaacagacttaatatttaaaactcttcttttaaatttaaccttaattcaacatcgaggtcataaatatttttttatatacgatctacaaaaaaatt	0.004	0.000	0.000	0.004	?
			Phoridae*	*Megaselia*_1*	gaattcaaaggtcgaacagacctaaactttaaacttctacacctaaaaataactcttaatccaacatcgaggtcgcaatcttttttgtcgatatgaactctaaaaaaaaa	0.004	0.000	0.000	0.004	−
			Psychodidae	Psychodidae_1	gaatttaaaagtcgaacagactttatttttaaactgctgcacctaaaataaatcttaattcaacatcgaggtcgcaatcatttttatcgatatgaactctctaaaaaga	0.000	0.004	0.000	0.004	−
		Hemiptera*	Aphididae*	Aphididae_1	gaatttaaaggtcgaacagacctaacacttaaaattttgcacctaagattaatcttaattcaacatcgaggtcgcaaactaatttttaaatttgaacttaaaaaattaa	0.004	0.000	0.000	0.004	?
				*Aphis gossypii**	gaatttaaaggccgaacagacctaatacttaaaattttgcacctaagattaatcttaattcaacatcgaggtcgcaaactaatttttaaatttgaacttaaaaaatgaa	0.004	0.000	0.000	0.004	+
			Cicadellidae	Cicadellidae_1	gaaattaaaagtcgaacagacttaaattataaagaatctaccccctaattttatcttaattcaacatcgaggtcgcaaactttaatatagatatgaactctccattaaaa	0.004	0.000	0.000	0.004	?
				*Nesophrosyne*_1	gattttaaaagtcgaacagacttaatataaagaattctgcttctaatataaatcttaattcaacatcgaggtcgtaaactttaatatagatatgaactccccattaaaa	0.004	0.000	0.000	0.004	−
		Hymenoptera*	NI	Hymenoptera_2	gattttaatagtcgaacagactaaataaattattatctccaataattattaatcttaattcaacatcgaggtcgcaaacttaaatatcaatatgatctctccatttaaa	0.004	0.000	0.000	0.004	?
			Aphelinidae	*Eretmocerus mundus*	aattttaatagtcgaacagactaaaatttttaatatcttcattaaaattaaattttaattcaacatcgaggtcgcaaacttttttatcaatatgaactatttaaaaaaa	0.004	0.000	0.000	0.004	?
			Apidae*	Apidae_1*	gattttaaaagtcgaacagacttaaatattaaactccttcatttaatttttatcttaattcaacatcgaggtcgcaaccacctttattaataggatctctctaaagata	0.022	0.000	0.000	0.022	?
				*Apis mellifera**	gactttaaaagtcgaacagacttaaatattaaactccttcatttaatttttatcttaattcaacatcgaggtcgcaatcacctttattaataagatctctctaaagata	0.009	0.000	0.000	0.009	−
			Braconidae	*Diaeretiella rapae*	gattttaaaagtcgaacagacttaaaatataaaaatttttcattttaattttatcttaattcaacatcgaggtcataaaatttattttaaatttgatcttagaaataaaa	0.009	0.009	0.000	0.018	+
			Formicidae*	Formicidae_2	gattttaatagtcgaacagactaaacttttaaacttcttcatttaaattaaatcttaattcaacatcgaggtcgcaaacatttttattaataagatctctccaaaaata	0.004	0.000	0.000	0.004	?
				Formicidae_3	gattttaaaagtcgaacagacttaaatattaaatatctccatttaatttttatcttaattcaacatcgaggtcgcaaacatttttatcaatatgatctttctaaaaata	0.004	0.000	0.000	0.004	?
				*Camponotus*_1	aattttaatagtcgaacagactaaaatattaaactcctacgcctaatttttattttaattcaacatcgaggtcgcaatcacttttatcaataagatctttctaaaaaga	0.000	0.000	0.004	0.004	−
				*Cardiocondyla emeryi*	gattttaaaagtcgaacagacttaaatattaaatttctccatctaatttttatcttaattcaacatcgaggtcgcaaacatttttatcaatatgatctctccaaaaata	0.004	0.000	0.000	0.004	?
				*Monomorium subopacum**	gattataaaagtcgaacagacttaaatattaaatatctccatttaatttttatcttaattcaacatcgaggtcgcaatcacttttatcaatatgaactctctaaaaata	0.013	0.000	0.000	0.013	−
			Thynninae	*Neozeleboria*_1	gattttaaaagtcgaacagacttaaatattaaacttctccatctaatttttatcttaattcaacatcgaggtcgcaaccatttttatcaatatgaactctccaaaaata	0.004	0.004	0.000	0.009	?
		Lepidoptera*	NI	Lepidoptera_1	gattttaatgatcgaacagatcaaaaattttaaacttttgcatttaaattttatcttagtccaacatcgaggtcgcaaacttttttttttatttgaactaaaaaaaaaaa	0.013	0.009	0.000	0.022	?
				Lepidoptera_2*	gattttaatgatcgaacagatcaaaaacttaaacttttgcattataaattttatcttaatccaacatcgaggtcgcaaactctttttcttataagaactaaaaaaaaaa	0.004	0.000	0.000	0.004	?
				Lepidoptera_3	gattttaatgatcgaacagatcaaaaattttaaacttttgcatttaaattttatcttagtccaacatcgaggtcgcaaacttttttttttatttgaactaaaaaaaaa	0.004	0.004	0.000	0.009	?
				Lepidoptera_6	gattttaatgatcgaacagatcaaaaattttaaacttttgcatttaaattttatcttagtccaacatcgaggtcgcaaacttttttttttatttgaactaaaaaaaaaga	0.000	0.004	0.000	0.004	?
				Lepidoptera_8	gattttaatgatcgaacagatcaaaattttaaacttctacatttaaattttatcttaatccaacatcgaggtcgcaaacttttcttcttatttgaactaaaaaaaaaa	0.004	0.000	0.000	0.004	?
			Bombycidae	*Bombyx*_1	gattttaatgatcgaacagatcaaaattttaaacttctacatttaaattttatcttaatccaacatcgaggtcgcaaacttttcttcttatttgaactaaaaaaaaaaa	0.004	0.000	0.000	0.004	?
			Noctuidae	Noctuidae_1	gattttaatgatcgaacagatcaaaattttaaacttctgcatttaaattttatcttaatccaacatcgaggtcgcaaactcttttttttatttgaactaaaaaaaaaa	0.004	0.000	0.000	0.004	−
			Papilionidae	Papilionidae_1	gattttaatgatcgaacagatcaaaaattttaaacttttgcatttaaattttatcttagtccaacatcgaggtcgcaaactttttttttatttgaactaaaaaaaaaaa	0.013	0.004	0.000	0.018	?
				Papilionidae_2	gattttaatgatcgaacagatcaaaaattttaaacttttgcatttaaattttatcttagtccaacatcgaggtcgcaaactttttttttatttgaactaaaaaaaaaa	0.009	0.004	0.000	0.013	?
			Pieridae	*Colotis*_1	gattttaatgatcgaacagatcaaaattttaaacttttgcatttaaattttatcttaatccaacatcgaggtcgcaaacttttatttttatttgaactaaaaaaaaaaa	0.004	0.000	0.000	0.004	−
			Pterophoridae	Pterophoridae_1	gattttaatgatcgaacagatcaaaattttaaatttttgcatttaaattttatcttaatccaacatcgaggtcgcaaacttttatttttatatgaactaaaaataaaaa	0.004	0.000	0.000	0.004	?
		Orthoptera	Acrididae	Acrididae_1	gaatttaaaggtcgaacagacctaatcattgggctactgcacccaaaatttttcttaatccaacatcgaggtcgcaatctgctttgtcgatatgagctctcaaaaacga	0.004	0.000	0.000	0.004	−
				Acrididae_2	gaatttaaaggtcgaacagacctaatcattgggctactgcacccaaaatttttcttaatccaacatcgaggtcgcaatctgctttgtcgatatgagctctcaaaaacaa	0.004	0.000	0.000	0.004	−
			Gryllidae	*Gryllus*_1	gaatttaatggtcgaacagaccaattaattaagcttctgcacctaataattttcttaatccaacatcgaggtcgcaatctttattatcaatatgaactctccaataaca	0.000	0.000	0.004	0.004	−
			Pyrgomorphidae	*Pyrgomorpha conica*	gattttaaaggtcgaacagacctagtttctaaactgatgcacctagaacttatcttaatccaacatcgaggtcgcaatctaatttgttgatatgaactctcaaaattaa	0.004	0.000	0.000	0.004	−
		Thysanoptera	Phlaeothripidae	Phlaeothripidae_1	gattttaaaagtcgaacagacttaatttattaaaatttttcttaaaaaatttatcttaattcaacatcgaggtcataaaaaatatattaaaataaggactttttatataatt	0.004	0.000	0.000	0.004	−
	Malacostraca	Decapoda	NI	Decapoda_1	aaatttaatggttgaacaaaccaacttactaggatcctgctcctaatagtaattttaattcaacatcgaggtcgcaacccactctattgatacggactcttcagagtga	0.004	0.000	0.004	0.009	?
				Decapoda_2	atatttattagtcgaacagactgtcttttataaactgctgcttctaaaagaaaatttaattcaacatcgaggtcgcaaaaacttttatcgatttgaactctccaaaagaa	0.004	0.000	0.000	0.004	?

Note

The final ID of MOTUs corresponds to the highest taxonomical classification possible. Information about the putative impact (positive, +, negative, −, or unknown, ?) of predation on each MOTU is also given. The (*) indicates taxa that contributed significantly to differences between sections.
